# On the effect of microwave energy on the Michael addition of dimethyl malonate on levoglucosenone

**DOI:** 10.1016/j.mcat.2024.114394

**Published:** 2024-08

**Authors:** Léa Charrier, C. Peter Howe, Maria Jose Calandri, Valeria Marisa Rocca, Con Robert McElroy, Alessandro Pellis

**Affiliations:** a University of Genova, Department of Chemistry and Industrial Chemistry, via Dodecaneso 31, 16146, Genova, Italy; b University of York, Department of Chemistry, Green Chemistry Centre of Excellence, YO10 5DD, Heslington, York, UK; c University of Lincoln, School of Chemistry, LN6 7DL, Lincoln, UK

**Keywords:** Levoglucosenone, Dimethyl malonate, Microwave irradiation, Sustainable synthesis, Platform molecules

## Abstract

Among biomass-derived platform molecules one of the most prominent structures is levoglucosenone (LGO) from which it is possible to derive a wide array of solvents, chemicals, and polymeric materials. In this work we investigated the Michael addition of dimethyl malonate on levoglucosenone by testing several alternative catalysts ranging from Lewis acids to structured silicas and clays. The work had the double aim to i) optimize the reaction using the widely reported KF/Alumina catalyst, giving a frame of reference for its relative activity in this Michael addition and ii) conduct a catalyst screen while investigating various reaction mechanisms. Among the tested catalysts, Ca(OH)_2_ was the best candidate to substitute KF/Alumina, reaching yields >90 % after only 5 min of microwave irradiation.

## Introduction

1,6-anhydro-3,4-dideoxy-β-D-glycero-hex-3-enopyranos-2-ulose, commonly known as levoglucosenone (LGO) is a cellulose-derived molecule with great potential as a key platform molecule leading the transition of industry and manufacturing away from crude oil towards developing a sustainable bio-economy [1]. LGO’s market value has been estimated to be worth in excess of ~70,000 times that of ethanol [2], which has the potential to challenge the market dominance of petrochemicals.

LGO, first identified in the 1970s, is an anhydro sugar derived primarily from glucose, that combines an α,β-unsaturated ketone (a ready electrophile, and a highly activated ketone) as well as a chiral acetal center. This makes it an attractive dienophile for Diels-Alder Reactions and a good Michael acceptor [3–5]. From a synthetic standpoint however, the most crucial component that gives LGO its greatest potential resides in the retention of one of the chiral centers of glucose, fixed in its glucosidic anhydro bridge [6]. LGO is naturally enantiopure as a result and has broad potential as a chiral synthon, especially important in the development of pharmaceuticals and bioactive compounds. The bridge also makes the faces of LGO physically and chemically different, allowing for high regio and stereo-selectivity in reactions.

To further expand the knowledge of Michael additions reactions onto bio-based building blocks, in this work we investigated the Michael addition of dimethyl malonate (DMM) on levoglucosenone (LGO) going beyond the available literature by testing several catalysts with the double aim of i) conducting a catalyst screen benchmarked against the widely reported activity of KF/Alumina (KF/Alu) [7] and ii) conduct a catalyst screen while investigating various reaction mechanisms.

### Lewis acids

**Fig. 1:**
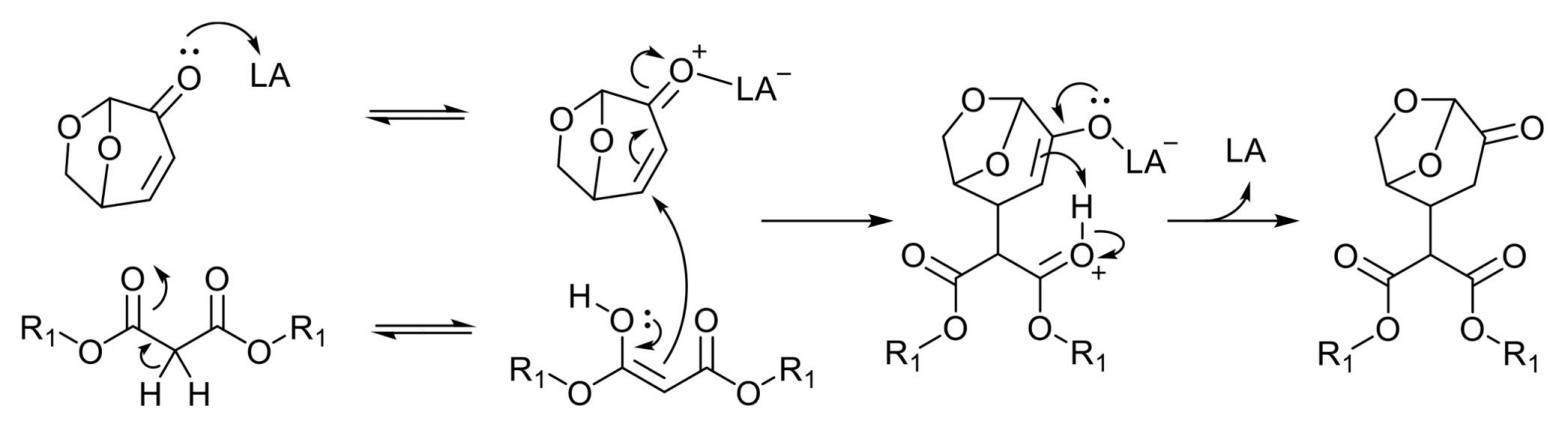
Mechanism of the acid-catalyzed Michael addition of DMM on LGO.

Although base mediated reactions are more commonly reported, Lewis acids have also been mentioned as successful catalysts in Michael additions [8]. In the DMM-LGO Michael reaction, Lewis acids will accept electrons from electron donors, such as oxygen atoms, forming an adduct that activates the compound (Fig. 1). In forming the Lewis-adduct, the Lewis acid lowers the energy level of the lowest unoccupied molecular orbital (LUMO) of the adjacent π-system [9], LGO’s conjugated α,β-unsaturated ketone system. This allows better overlap between it and the highest occupied molecular orbital (HOMO) of the DMM, initiating the formation of the Michael adduct.

**Fig. 2:**
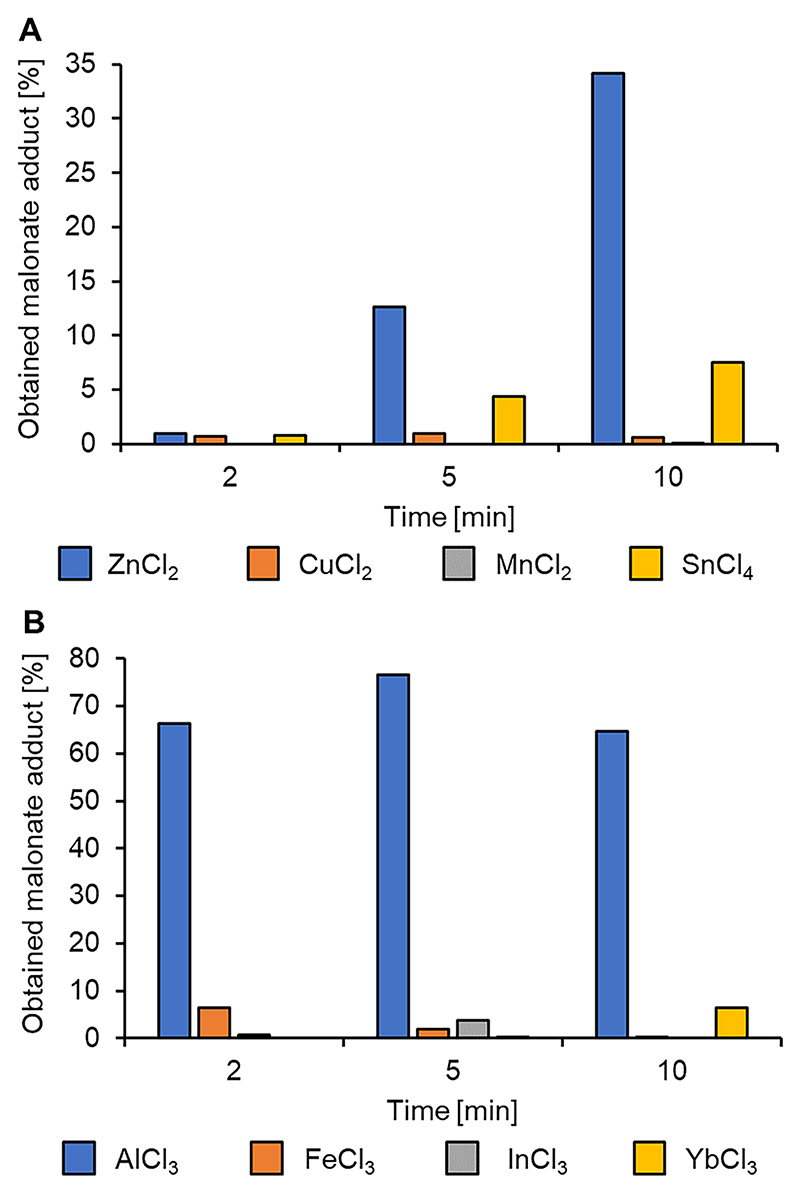
Lewis’s acid catalysts screened for the Michael addition of DMM to LGO.

The DMM-LGO Michael addition results summarized in Fig. 2 indicate that in general, Lewis’ acids were not particularly effective, leading to yields between 7 and 76 %. For most of the selected Lewis acid catalysts (CuCl_2_, MnCl_2_, FeCl_3_, InCl_3_ and YbCl_3_) the reaction either did not proceed or gave conversions below 10 %. Some interesting activity was observed using ZnCl_2_ (Fig. 2a), achieving up to 90 % LGO conversion, but with only 30 % selectivity towards the desired DMM-LGO adduct. The exception was AlCl_3_, which achieved a 76 % yield to the DMM-LGO adduct. This conversion was observed after a 5 min MW irradiation time at 10 W (Fig. 2b), after which selectivity towards DMM-LGO decreased as unwanted side reactions started to take place.

All these Lewis acids readily dissolve in the reaction solution and therefore act as homogeneous catalysts. This makes recovery and separation of the reaction product more difficult when compared with e.g. heterogeneous KF/alumina catalyst. In conclusion, Lewis acids are not effective catalysts for this Michael addition since, in order for them to compete in terms of sustainability, they would need to be significantly more effective boosting conversion, shortening reaction time and improving selectivity.

### Brønsted acids

The Brønsted acid catalyzed Michael addition in theory works similarly to the Lewis acid mechanism, activating the LGO by protonating the carbonyl oxygen.

**Fig. 3:**
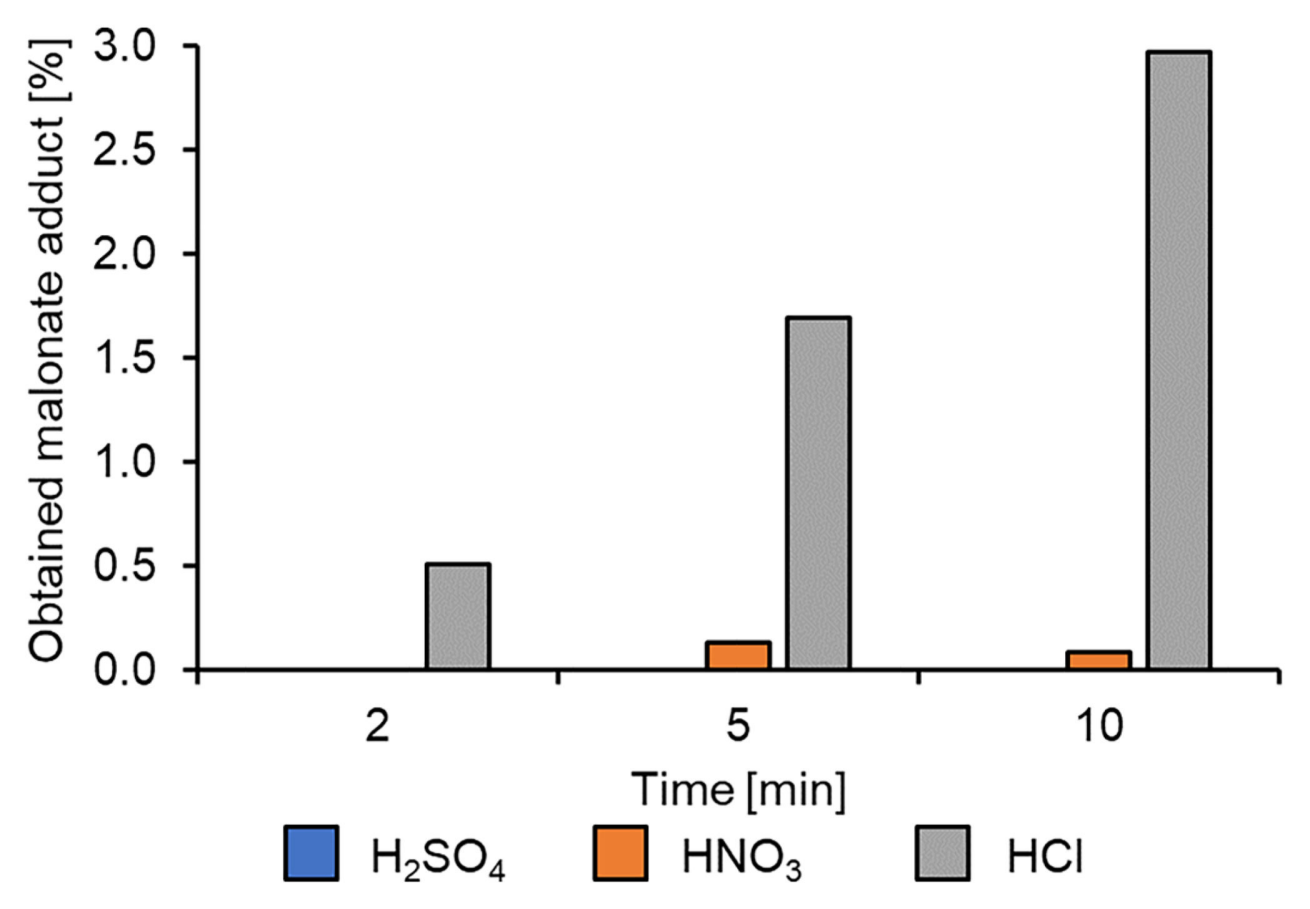
Brønsted acid catalysts screened for the Michael addition of DMM to LGO.

Three Brønsted acids were chosen to conduct the screen: sulfuric acid, nitric acid, and hydrochloric acid. The results, shown in Fig. 3, were underwhelming. Nitric acid shows limited yield, while HCl catalyzed reactions produced several byproducts but only a 3 % yield of the desired LGO-DMM adduct. In fact, in the HNO_3_ catalyzed reaction, all the DMM was consumed while 20 % unreacted LGO still remained. H_2_SO_4_ also failed to catalyze the desired Michael addition, yielding a black sponge-like insoluble material with no product detected.

### Bases

**Fig. 4:**
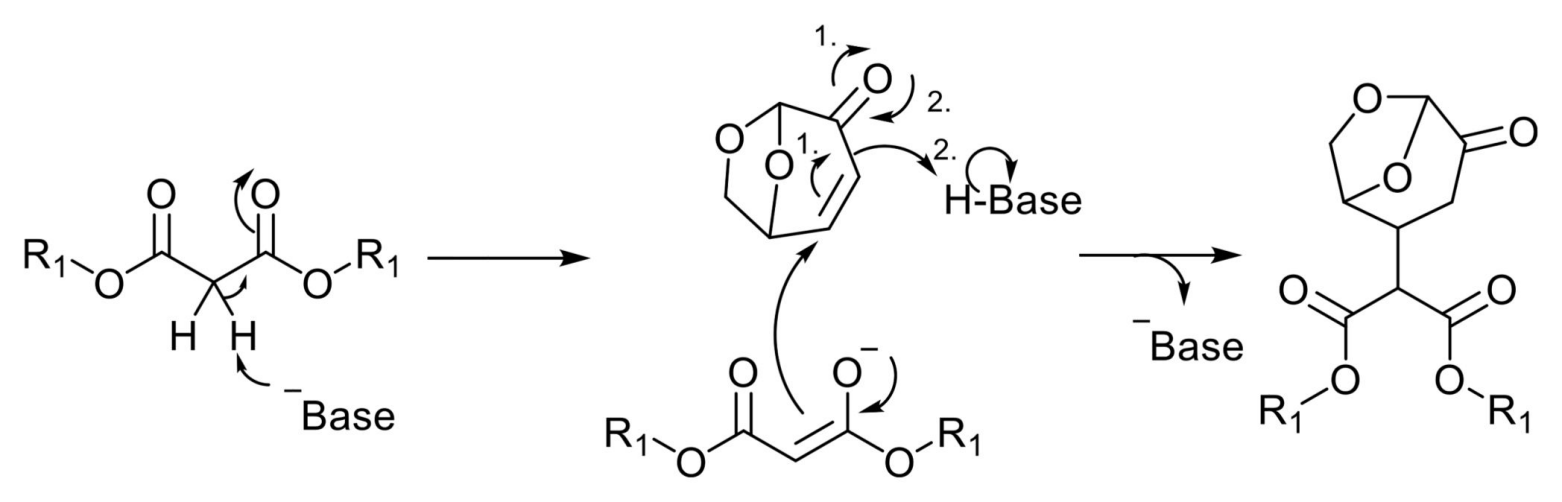
Mechanism of the base-catalyzed Michael addition of DMM on LGO.

For Michael addition reactions, a significative difference between Lewis and Brønsted acids can be observed as the Lewis base-adduct is not able to activate LGO. For this reason, in the next instance, we tried several bases as potential catalysts for this reaction. The proposed mechanism is that a Brønsted base is generated in situ by reacting with atmospheric water. KF/Alumina for example is a well-known catalyst employed in Michael reactions on other substrates. Brønsted base generation is likely to explain its activity toward this reaction (Fig. 4).

**Fig. 5:**
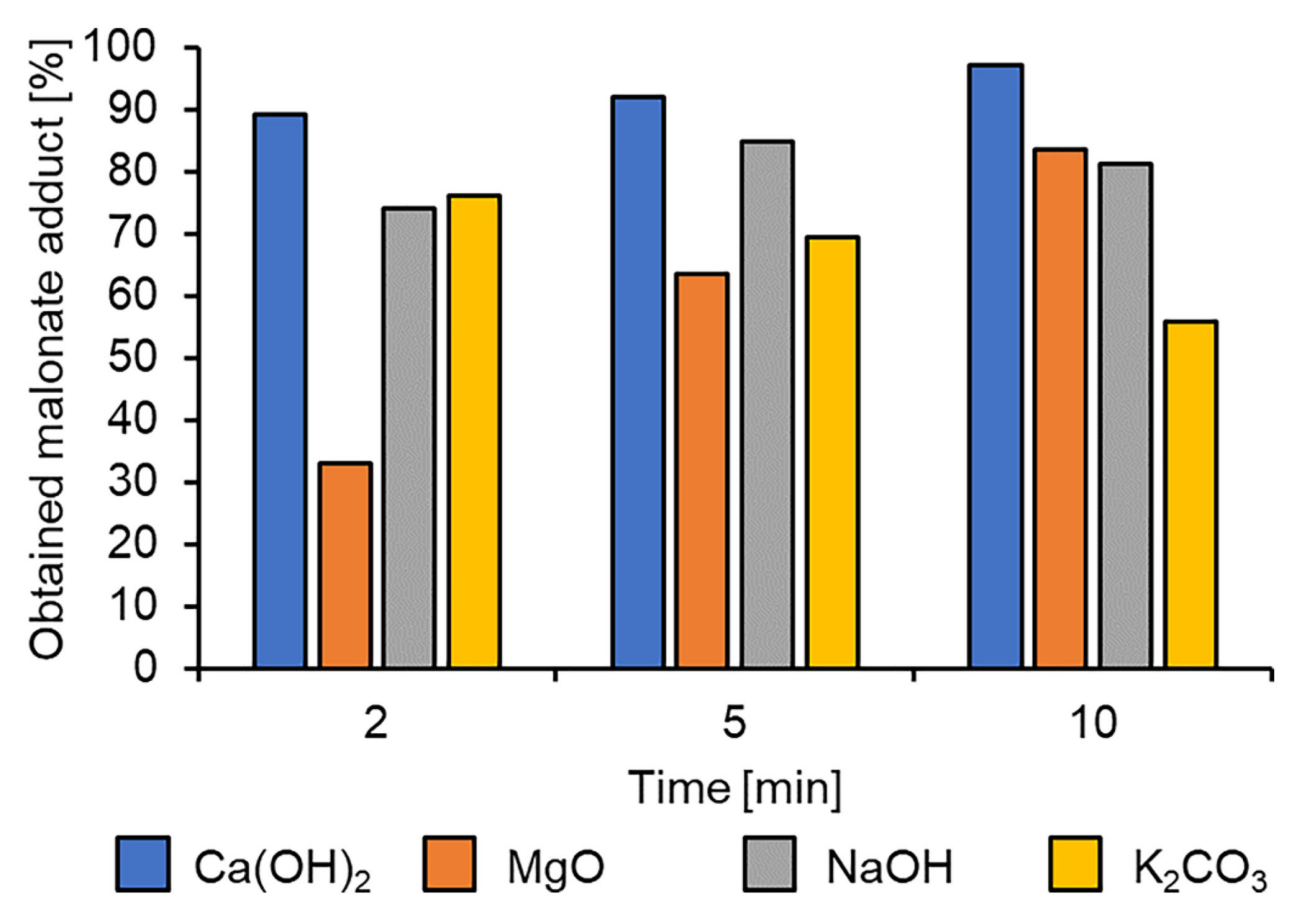
Base catalysts tested for the Michael addition of DMM to LGO.

A selection of four bases were tested in the Michael addition with levoglucosenone: the heterogeneous solid Lewis bases calcium oxide and magnesium oxide, the solid Brønsted base potassium carbonate and a two molar aqueous solution of sodium hydroxide (Fig. 5). Upon initial screening reasonable to good yields were observed with stock historical catalysts. When repeated with newly bought catalysts, reaction yields were in broad agreement except for CaO which showed no activity. XRD data showed that the historical CaO had hydrolyzed to Ca(OH)_2_ which is the catalytically active species. In contrast, MgO showed little to no evidence of hydrolysis to the respective hydroxide. As such Ca(OH)_2_ was used in place of CaO for this research.

Base screening was much more successful than the acids, which is again consistent with KF/alumina, a base, successfully catalyzing the reaction. Among the tested bases we observed a noticeable variation in their catalytic activity. Magnesium oxide (Fig. 5, orange bars) lead to a maximum yield of 84 % after 10 min of MW irradiation while calcium hydroxide (Fig. 5, blue bars) reached a 91 % yield after 5 min that further improved to 97 % after 10 min.

**Fig. 6:**
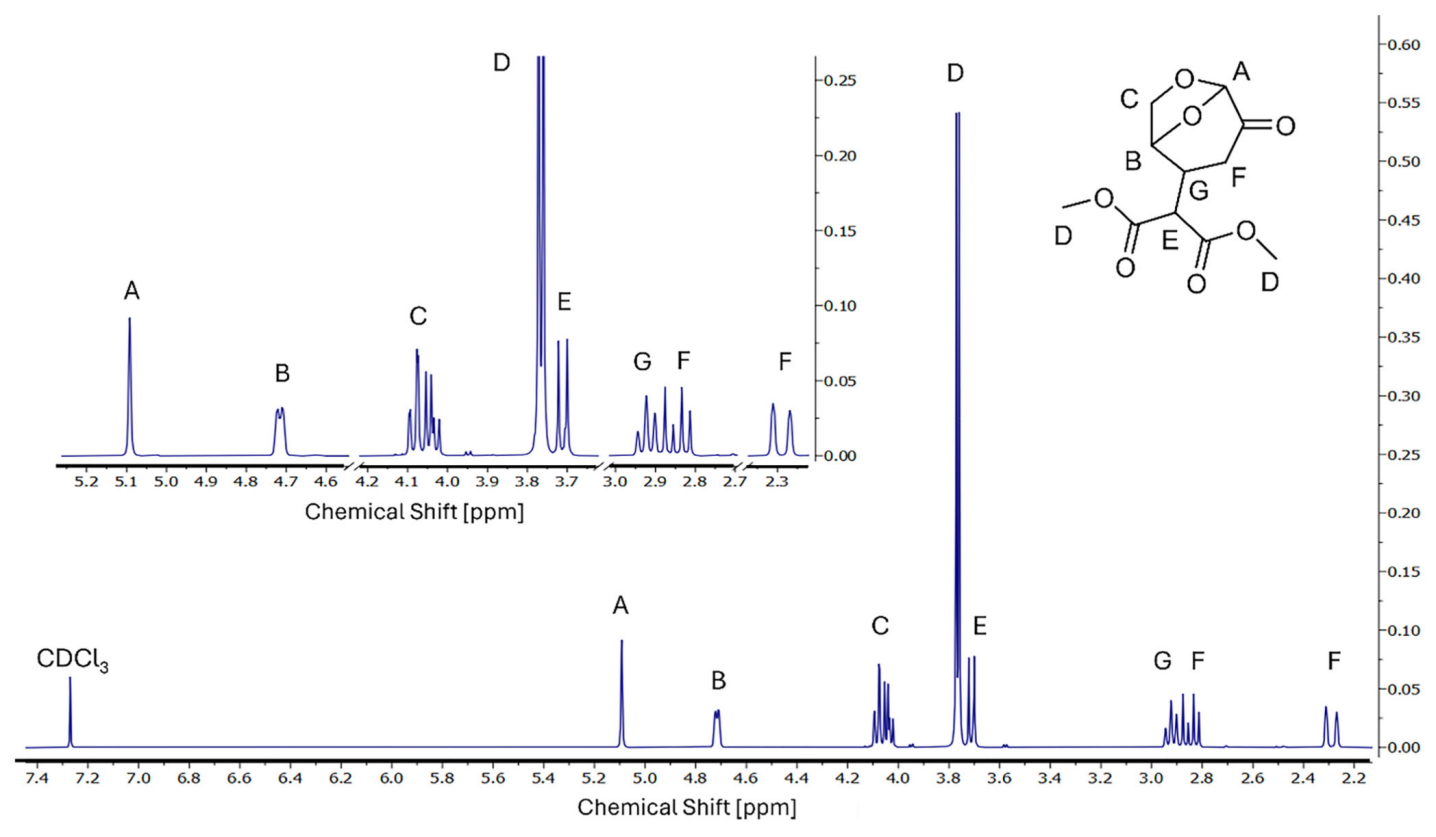
^1^H NMR of the reaction product obtained when reacting DMM with LGO using Ca(OH)_2_ as the catalyst for 10 min under a constant irradiation of 10 W.

While the Brønsted bases achieved yields in the 75–80 % range, Ca (OH)_2_ seems to exceed the catalytic ability of KF/alumina to catalyze this kind of Michael addition (see section Comparison of the KF/alumina and Ca(OH)_2_-catalyzed reactions). The fully assigned ^1^H NMR spectra of the reaction product shown in Fig. 6 together with the LC-MS results (see ESI, Fig. S1) further confirm that the obtained reaction product has the desired structure and, when reacting DMM and LGO in a 1:1 ratio, a high purity (>95 %) even without having to go through a long and tedious column chromatography purification process.

**Fig. 7:**
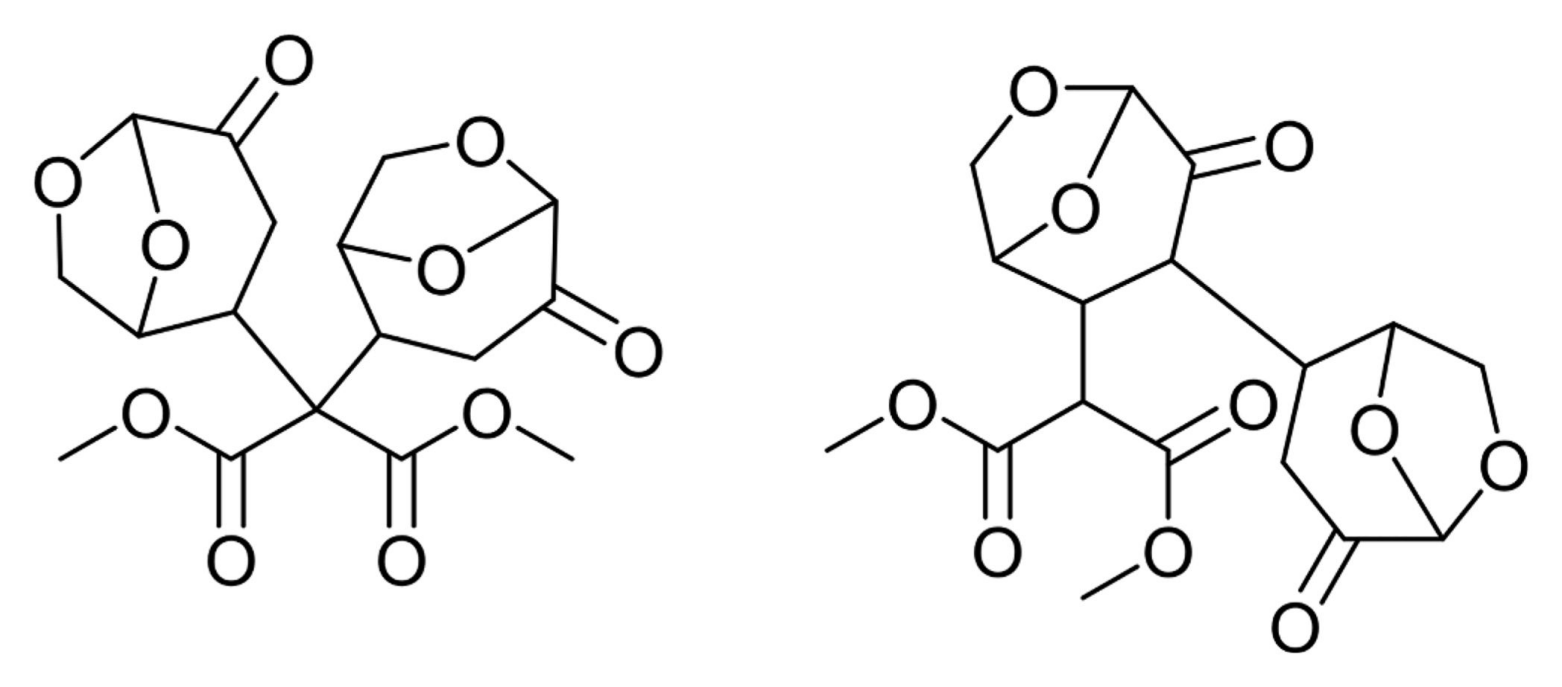
Potential structures of the side reaction products of the base-catalyzed reaction between LGO and DMM. Both isomers have a calculated *m/z* value of 385 corresponding to the signal found via LC-MS analysis.

It is interesting to note that both the 2 M sodium hydroxide and the solid potassium carbonate reach yields >70 % after only 2 min of MW irradiation. The yield obtained using sodium hydroxide does improve to 84 % after 5 min (while the K_2_CO_3_ decreases), but after 10 min of irradiation both catalysts lead to a decrease of the desired reaction product with K_2_CO_3_ dropping from a high of 76 % obtained after 2 min to 55 % at 10 min and with the 2 M NaOH reaching a plateau at ~80 % yield. One possible explanation for observing this decreasing trend is that the strength of the bases is inducing a retro-Claisen reaction in the DMM. The GC analysis did not indicate the presence of any smaller organic compounds in comparison to DMM and LGO but instead a couple of other compounds with retention times greater than either starting material were observed. The main side product has a retention time of 7.060 min. If indeed the retro-Claisen reaction is occurring, the resultant chemicals are likely reacting with the starting materials to form larger molecules. To confirm that a GC–MS analysis of the reaction product was carried out and a molecular ion of 385 *m/z* for the compound at 7.060 mins was identified (see ESI, Fig. S2). This mass is equivalent to two isomers: the double Michael addition adduct, and the Michael adduct between DMM and the LGO dimer (Fig. 7). This base-catalyzed reaction might even bring to the formation of even larger oligomeric species as previously reported by Shafizadeh and collaborators [4].

### Structured silicas

The structured silica SBA-15 and specifically its amino-propyl functionalized variant, amino SBA-15 were chosen as part of the screen, because they were previously reported to be effective at catalyzing Michael addition reactions [10]. As a heterogeneous base catalyst, the mechanism for the amino-propyl functionalized surface would proceed like a conventional base (see Fig. 4), with the added benefit of adsorption onto the catalyst surface stabilizing the reactants and lowering the activation energy.

**Fig. 8:**
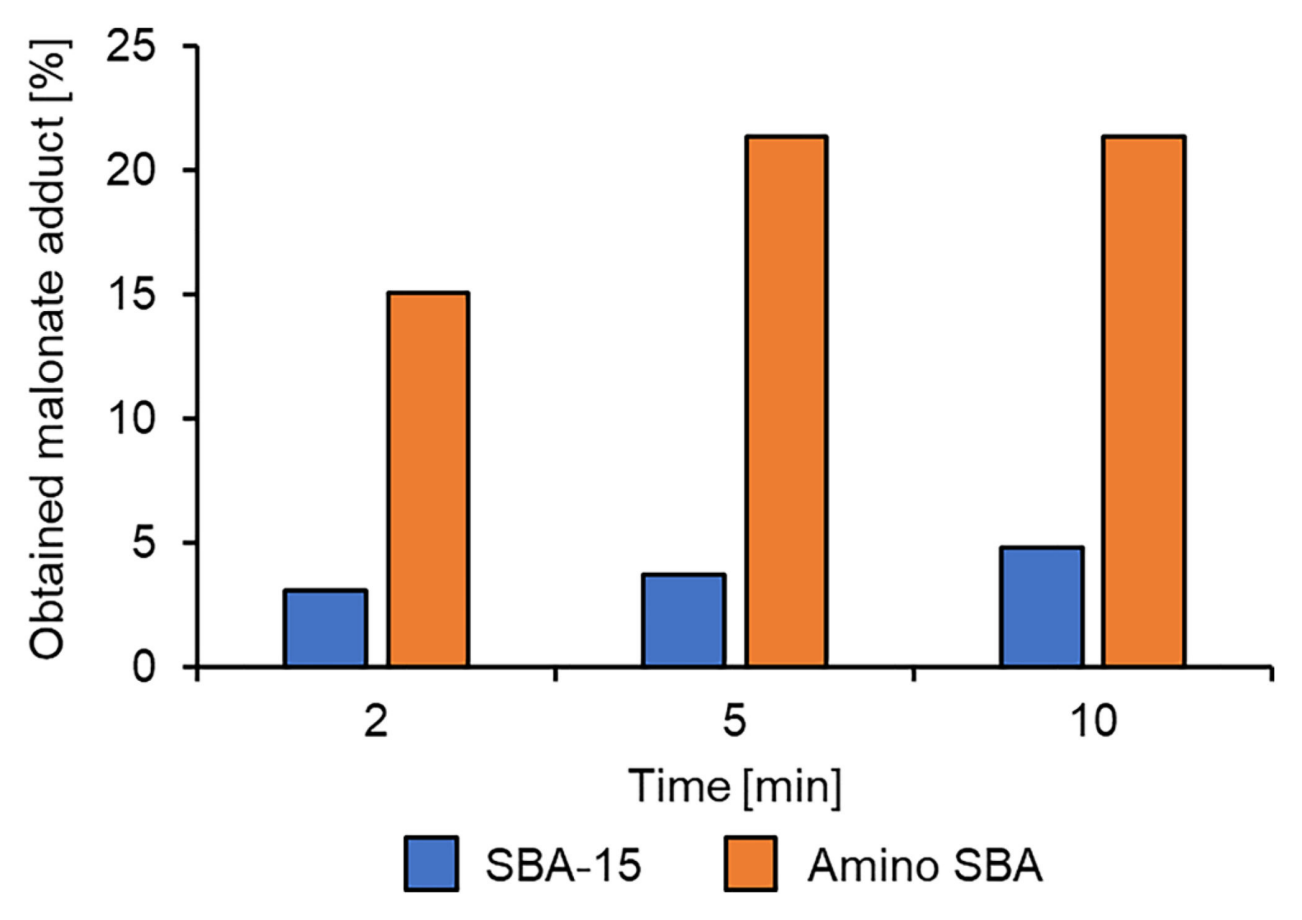
Structured silicas tested for the Michael addition of DMM to LGO.

To understand the interaction of the mesoporous silica with the reactants in the reaction the unfunctionalized SBA-15 silica was also run as a control. The results indicated that SBA-15 has some slight catalytic activity in the reaction (Fig. 8, blue bars), but reached a maximum yield of only 4.9 %. The amine functionalized SBA-15 did display noticeably better catalytic ability, reaching a 21 % yield after a 5 min MW irradiation.

It is interesting to note that the amino SBA-15 more readily enabled the catalysis of an undesired side reaction with the major byproduct having a selectivity of 69 % after 10 min (overall conversion of 92 % including the desired Michael adduct). This product, having a GC retention time lower than the DMM-LGO Michael adduct (4.300 min vs 6.230 min) was identified as the LGO-MeOH adduct as the compound has a molecular weight of 158 g·mol^−1^, compared to the DMM-LGO Michael adduct which has a molecular weight of 258 g mol^−1^ (see ESI, Fig. S3). As no methanol was used in the reaction, is it likely generated in- *situ* by a proton being abstracted from atmospheric water by the basic surface, generating a hydroxide ion that can readily hydrolyze the DMM adsorbed onto the immediately surrounding surface.

### Clays

Due to the fact that Lewis acids are typically used as homogeneous catalysts, in order to heterogenized them and thus make removal and purification of the product easier and more resource efficient, montmorillonite (Mont) and montmorillonite K10 (K10) were chosen to stabilize the metal center [11]. The negatively charged silicate layers of the clay can coordinate with the metal ion, while broadly maintaining catalytic ability in aqueous or solvent-free conditions.

**Fig. 9:**
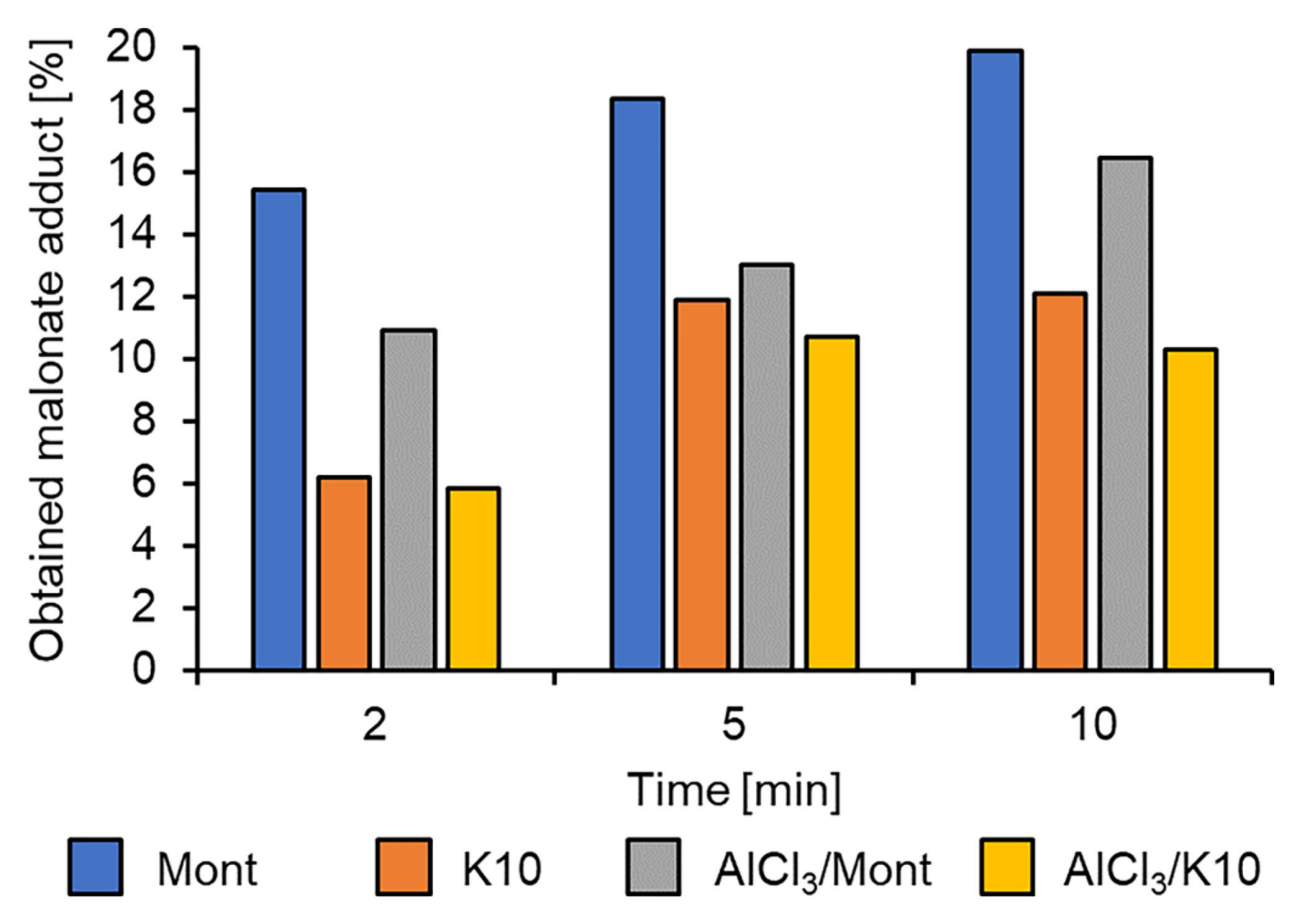
Clays tested for the Michael addition of DMM to LGO.

**Fig. 10:**
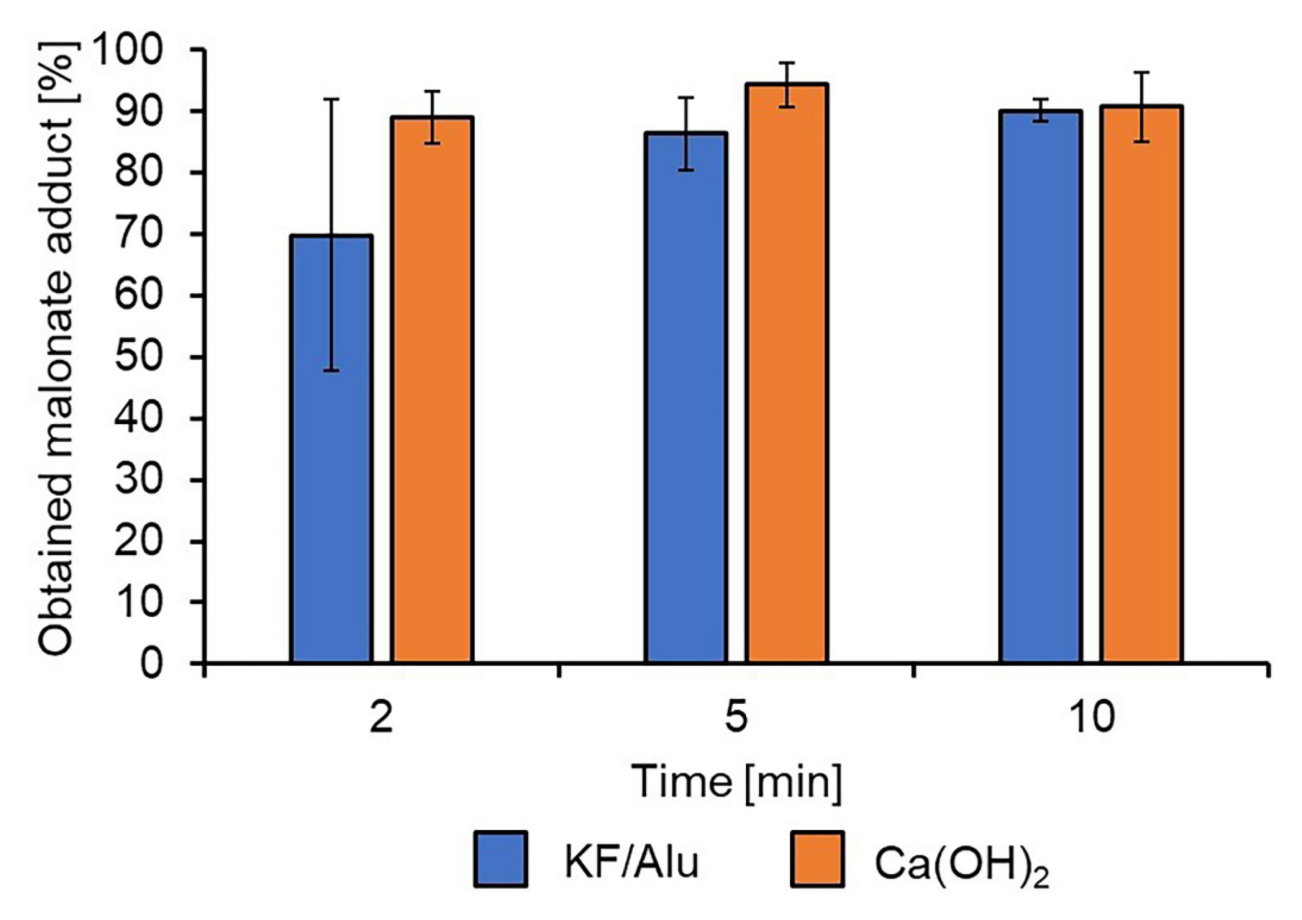
Comparison of Ca(OH)_2_ and KF/Alu as the catalyst for the Michael addition of DMM.

Aluminium chloride (AlCl_3_) was the only Lewis acid in the screen that displayed any significant catalytic ability in the Michael addition, so it was the only Lewis acid tested in the ion exchanged clays. For comparison, the reactions were run with the unaltered clays, as well as the ion exchanged variants, to quantify the role of the clay surfaces themselves in relation to the Lewis acid. All the clays achieved the best conversions (10–20 %) to the desired DMM-LGO adduct after a 10 min MW irradiation using a fixed power of 10 W. The ion exchanged clays (Fig. 9, grey and yellow bars) performed worse than their unaltered counterparts in both cases (Fig. 10, blue and orange bars). For instance, Mont reached a yield of 20 %, while AlCl_3_/Mont achieved a best yield of 16 %. Regarding potential secondary reactions, total LGO conversions of 24 % and 19 % were observed for Mont and AlCl_3_/Mont respectively, therefore showing a very limited presence of unwanted side products.

### Comparison of the KF/alumina and Ca(OH)_2_-catalyzed reactions

Of all the catalysts screened in this work, the one that showed the most promising results was calcium hydroxide, yielding to conversions similar -and sometimes exceeding- those of the well-known KF/alumina [7]. To determine which was the most effective both reactions were repeated three times to assess the reproducibility of the reactions.

Overall, the two bases have a very similar activity in catalyzing the DMM-LGO Michael reaction. After 2 min of MW irradiation both are widely inconsistent simply because of the varied states of heating and mixing in the reaction vessel but achieve a reproducible, high conversion (>90 %) and tight distribution between reaction at higher reaction times. Ca(OH)_2_ is rather fast to start the addition reaction and does appear to reach near maximum conversion after 5 min of MW irradiation at 10 W, yielding an average Michael adduct yield of 94 % (Fig. 10, orange bars), compared to KF/Alu reaching the maximum yield (90 %) after 10 min (Fig. 10, blue bars). Of note, 50 mg of Ca(OH)_2_ equates to a loading of 27 mol% with relation to LGO. The catalyst loading for KF/Alumina is 23 % by weight (KF to alumina support), as such 50 mg translates to an 8 mol% loading with relation to LGO. This suggests that KF/Alumina appears more catalytically active under these conditions with a turnover number of 11.3 compared to Ca(OH)_2_ with a turnover number of 3.5.

## Conclusions

From all tested catalysts (Lewis and Brønsted acids, bases, structured silicas and clays), the best results in terms of selectivity and maximum conversions were obtained using Ca(OH)_2_ as the catalyst. On average yield values of 94–95 % were achieved using this mild base that effectively competes with the more widely used and reported KF/Alu catalyst. Due to the high catalytic efficiency and selectivity of both these catalysts in fact, other factors would likely decide the best candidate for further works on the topic such as production costs and their environmental impact.

## Supplementary Material

ESI

## Data Availability

Data will be made available on request.
