# Supplementary material for: On the effect of microwave energy on the Michael addition of dimethyl malonate on levoglucosenone
Source: Mol Catal. Author manuscript; Available in PMC 2024 Sep 18. (PMC7616458; doi:10.1016/j.mcat.2024.114394)
Supplement: ESI [file EMS198574-supplement-ESI.docx]

**Electronic Supplementary Information**

On the effect of microwave energy on the Michael addition of dimethyl malonate on levoglucosenone

Léa Charrier^1,λ^, C. Peter Howe^2,λ^, Maria Jose Calandri^1^, Valeria Marisa Rocca^1^, Con Robert McElroy^2,3,*^, Alessandro Pellis^1,*^

^1^ University of Genova, Department of Chemistry and Industrial Chemistry, via Dodecaneso 31, 16146, Genova, Italy

^2^ University of York, Department of Chemistry, Green Chemistry Centre of Excellence, YO10 5DD, Heslington, York, UK

^3^ University of Lincoln, School of Chemistry, LN6 7DL, Lincoln, UK

^λ^ These authors equally contributed to the work.

*Correspondence to: Dr. Con R. McElroy, email: [cmcelroy@lincoln.ac.uk](mailto:cmcelroy@lincoln.ac.uk) and Dr. Alessandro Pellis, email: [alessandro.pellis@unige.it](mailto:alessandro.pellis@unige.it)

**Materials and methods**

**Chemicals & catalysts**

Levoglucosenone was a kind gift from the Circa group. Potassium Fluoride was purchased from Alfa Aesar. Tetraethyl orthosilicate was acquired from Fluka. All other catalysts, chemicals and solvents were purchased from Sigma-Aldrich and used as received if not otherwise specified.

**Catalysts Used**

Lewis acids: boron trifluoride, aluminium chloride, Iron chloride, zinc chloride, tin chloride, copper chloride (Acros Organics), manganese chloride, indium chloride and ytterbium chloride. Brønsted acids: sulfuric acid, nitric acid, and hydrochloric acid. Bases: calcium oxide (Fluka), magnesium oxide (Alfa Aesar), potassium carbonate (Acros Organics) and 2 M aqueous solution of sodium hydroxide. Clays: montmorillonite (Mont) and montmorillonite K10 (K10).

**Catalysts preparation**

***Potassium Fluoride on Alumina (KF/Alu)***

Potassium Fluoride on Alumina (KF/Alu) was prepared according to the literature using a loading of 5 mmol g^-1^ KF^1^. Briefly, 75 mg of KF, 250 mg of alumina and 125 mL of methanol were mixed for 30 min at 50 ºC. The methanol was then evaporated under reduced pressure to yield the solid KF/Alumina catalyst.

***Aminopropyl SBA-15***

Aminopropyl SBA-15 was prepared according to the literature with some modification^2,3^. 8 g of Pluronic P-123 and 60 mL of deionized water were placed in a 1 L round-bottom flask. 240 mL of 2 M hydrochloric acid was then added, and the mixture was stirred at 40 ºC for 4 hours. 17 g of tetraethyl orthosilicate was then added dropwise and the mixture was left to stir at 40 ºC for a further 24 hours. The stirring was then stopped, and the temperature increased to 100 ºC for 24 hours. The SBA-15 was isolated by filtration and washed four times with 100 mL deionized water. The resulting product was then dried under vacuum for 6 hours and calcined at 550 ºC (ramp rate of 2 ºC min^-1^) for 5 h.

To functionalize the Aminopropyl SBA-15, 0.6 g of SBA-15 and 3 g of (3-aminopropyl)triethoxysilane were added to a round-bottom flask with 150 mL of Toluene. The mixture was heated to 60 ºC for 24 hours. The solid was filtered, washed using a toluene/ethanol mixture and then dried at 60 ºC until constant weight.

***Ion Exchanged Clays***

Ion exchanged Mont and K10 were prepared according to the literature^4^. 3 g of the clay was added to 100 mL aqueous solution containing 200 mg of AlCl_3_. The mixture was then heated to 80 ºC and stirred for 24 h. The clay was isolated by vacuum filtration using grade 1 Whatman filter paper and washed with deionized water, then dried at 110 ºC for 4 h before further use.

**Michael addition protocol**

For each reaction, a 10-mL CEM Discover microwave vessel was charged with 2.5 mmol of LGO, 6 mmol of DMM and 50 mg of the chosen catalyst. The reactions were conducted using a fixed microwave power of 10 W over periods of 2, 5 and 10 min using a CEM Discover microwave (CEM Corp., USA). The catalyst was filtered off and the reaction products analyzed without further purification steps.

**Gas Chromatography with Flame Ionization Detector (GC-FID)**

Reactions were monitored using gas chromatography coupled with a flame ionization detector (GC-FID). Chromatograms were obtained using an Agilent 6890N/Hewlett Packard HP 6890 series Gas Chromatography system fitted with a fused silica Restek Rxi-5HT column of 30 m length, 0.25 mm internal diameter and 0.25 μm film thickness. The GC-FID was operated using the following method: injection temperature: 300 °C, split ratio: 30:1, carrier gas: helium, gas flow: 2.0 mL min^-1^, injected volume: 1 μL, temperature ramp: 50 °C hold for 1 min, 50-300 °C linear ramp at 30 °C/min and hold at 300 °C for 5 min, detector temperature: 440 °C.

**Gas Chromatography with Flame Ionization Mass Spectrometer**

Products structures were elucidated via gas chromatography with flame ionization mass spectrometer (GC-FI MS) using a Waters GCT Premier TOF MS coupled to an Agilent 7890A GC system. The GC was fitted with a column with 30 m length, 0.25 mm internal diameter and 0.25 μm film thickness. The GC-FI MS was used with the following method: injection temperature: 280 °C, split ratio: 2:1, carrier gas: helium, gas flow: 2.0 mL min^-1^, injected volume: 1 μL, temperature ramp: 100 °C hold for 1 min, 100-300 °C linear ramp at 15 °C min^-1^ and hold at 300 °C for 5.67 min, detector temperature: 300 °C.

**Nuclear Magnetic Resonance (NMR) spectrometry**

^1^H and proton-decoupled ^13^C nuclear magnetic resonance (NMR) spectra were recorded on a JEOL ECS 400 MHz spectrometer. Samples were prepared by dissolving a small quantity of analyte in deuterated solvent, typically CDCl_3_ or D_2_O. Chemical shifts are reported in part per million (ppm) relative to tetramethylsilane.

**Fourier Transform Infrared (FT-IR) spectroscopy**

Functional groups were confirmed using Fourier Transform Infrared Spectroscopy (FT-IR), using a Perkin Elmer Spectrum 400 using a resolution of 1 cm^-1^.

**LC-MS**

The LC-MS Spectra were recorded on a Microsaic 4000MiD mass spectrometer connected with Agilent 1100 HPLC. Injected samples concentration was between 300-700 µg mL^-1^ Eluent was a 1:1 ultrapure water / acetonitrile mixture with 0.1% formic acid. Mass spectra were recorded in fullscan with a 100-800m/z mass range, positive mode and a Tic Voltage of 750V. HPLC was equipped with a Phenomenex Gemini C18 column 150x3mm 5 µm with a flow rate of 0.5 mL/min, temperature of 25°C, volume injection 5 µL and a ultrapure water/acetonitrile with 0.1% formic acid mobile phase gradient as follows: 0 min water with 0.1% formic acid= 90%, 20 min water with 0.1% formic acid =0%).

**
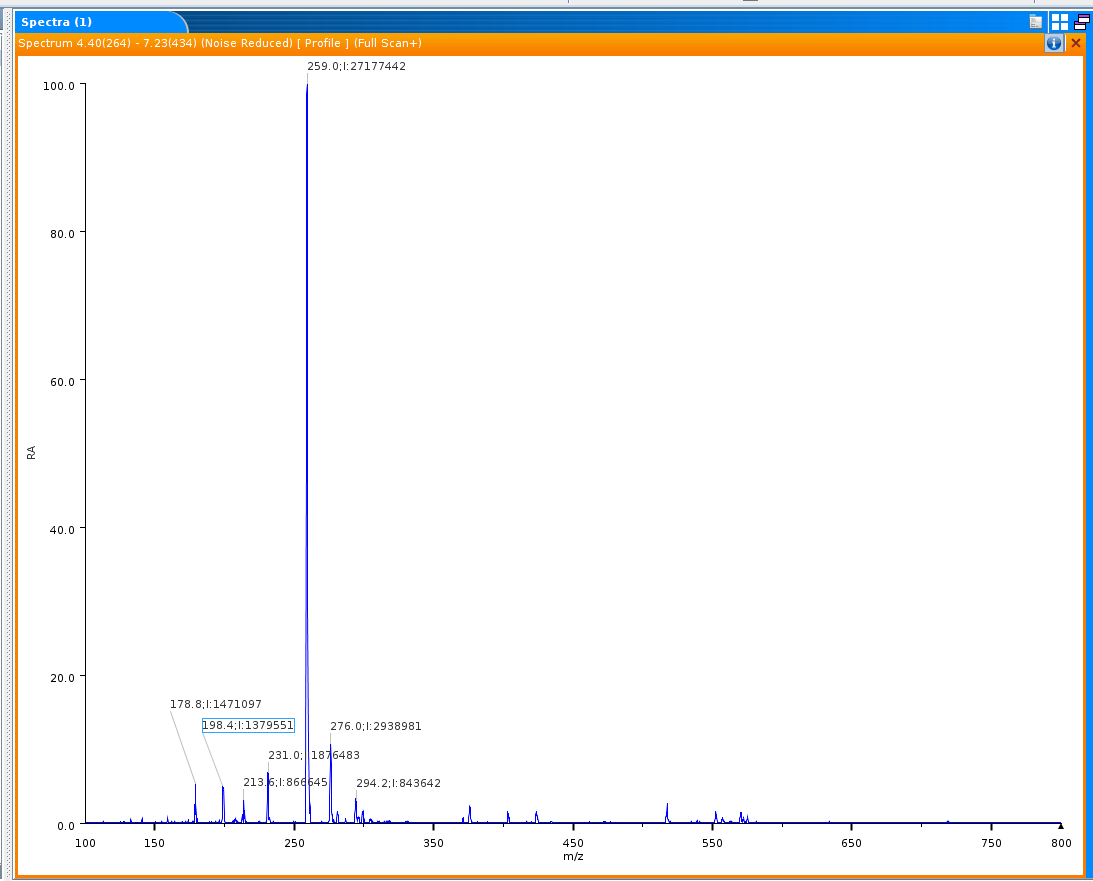
Figure S1.** LC-MS spectra of the main reaction product obtained when reacting DMM with LGO using Ca(OH)_2_ as the catalyst for 10 min under a constant irradiation of 10 W.


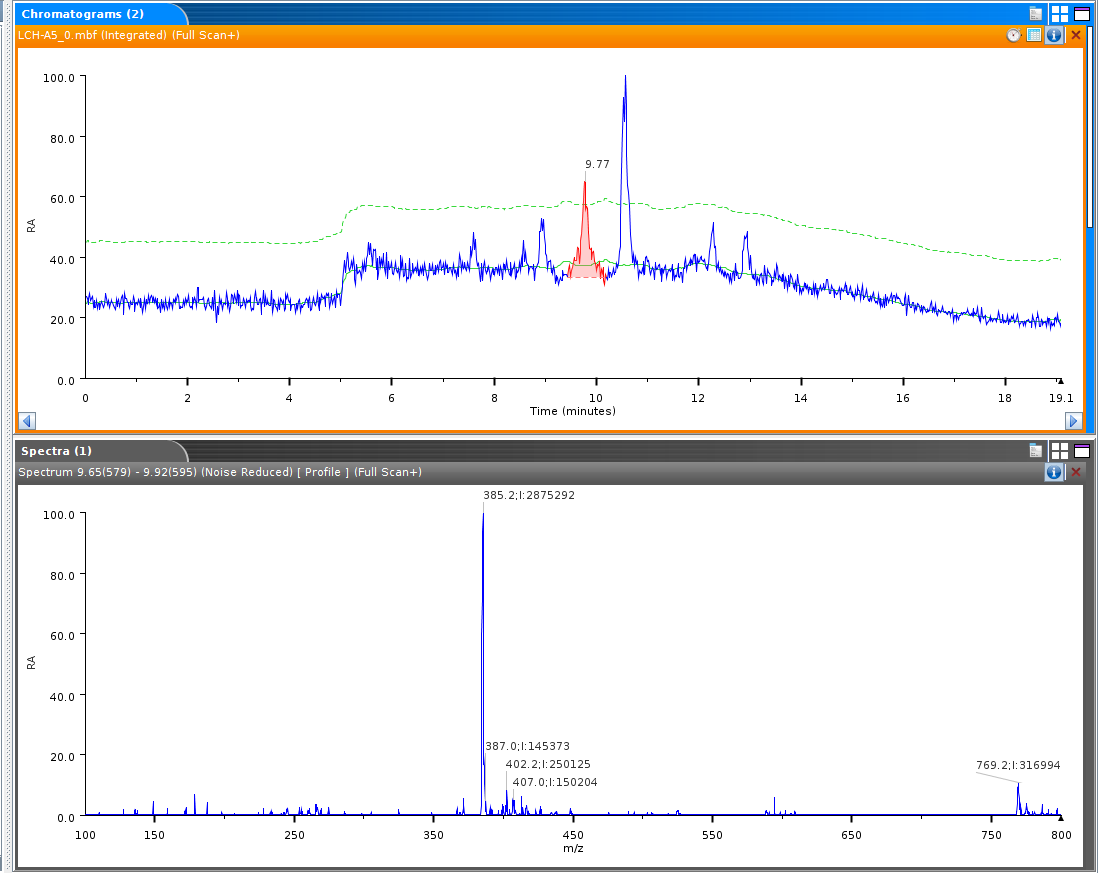
**Figure S2.** LC-MS spectra of the main reaction byproduct obtained when reacting DMM with LGO using NaOH as the catalyst for 10 min under a constant irradiation of 10 W.


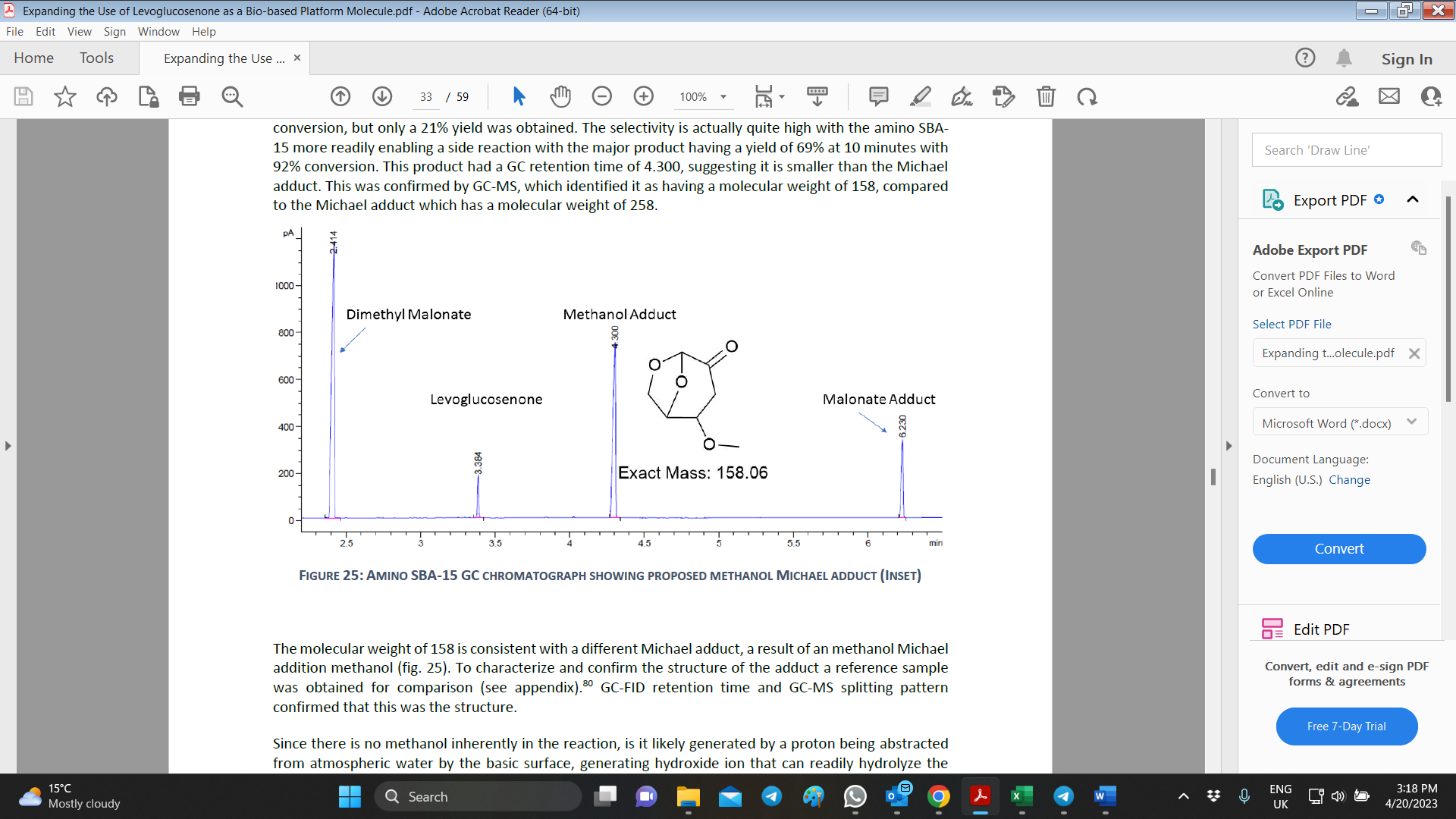


**Figure S3.** GC-FID spectra of the reaction product obtained when reacting DMM with LGO using SBA-15 as the catalyst for 10 min under a constant irradiation of 10 W.

**
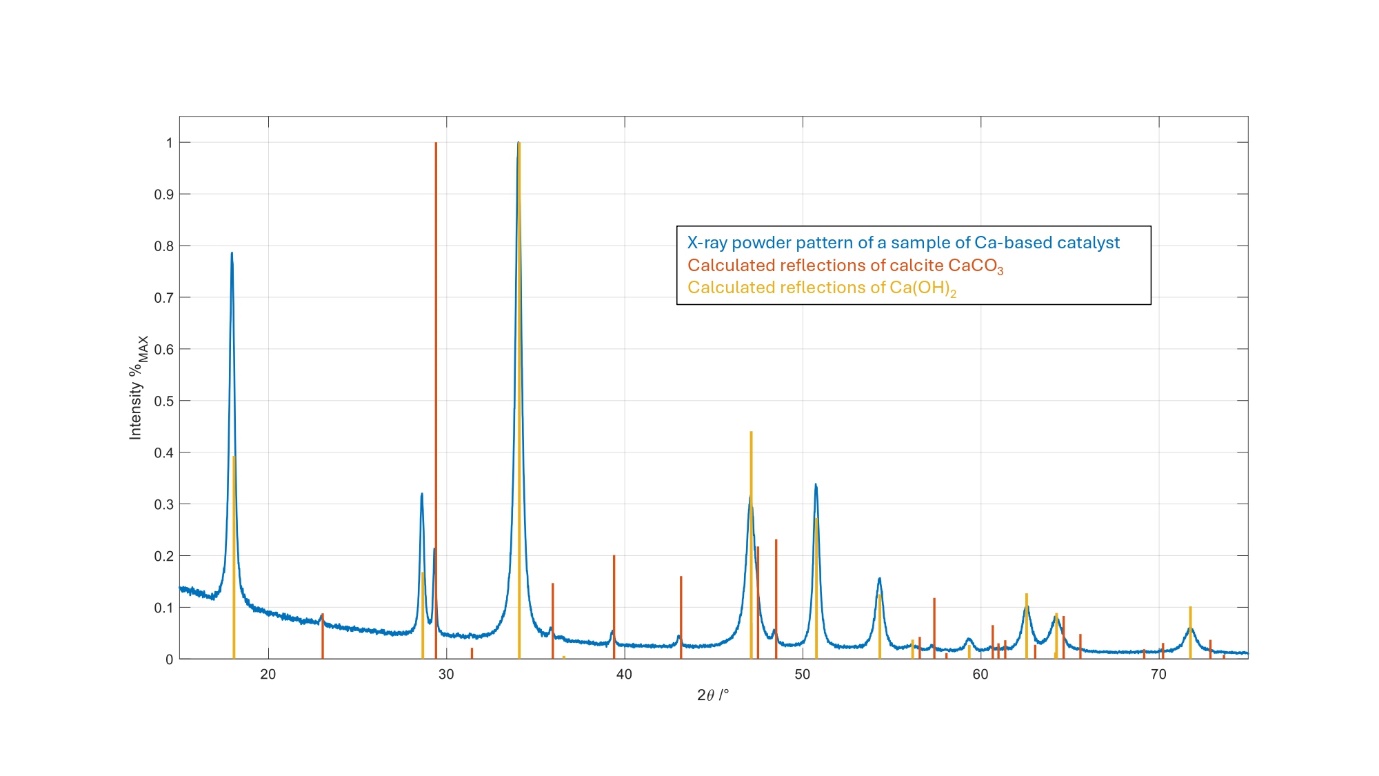
****Figure S4.** XRD analysis of the used CaO (blue line) compared with the calculated reflections of Ca(OH)_2_ (yellow line) and CaCO_3_ (red line).


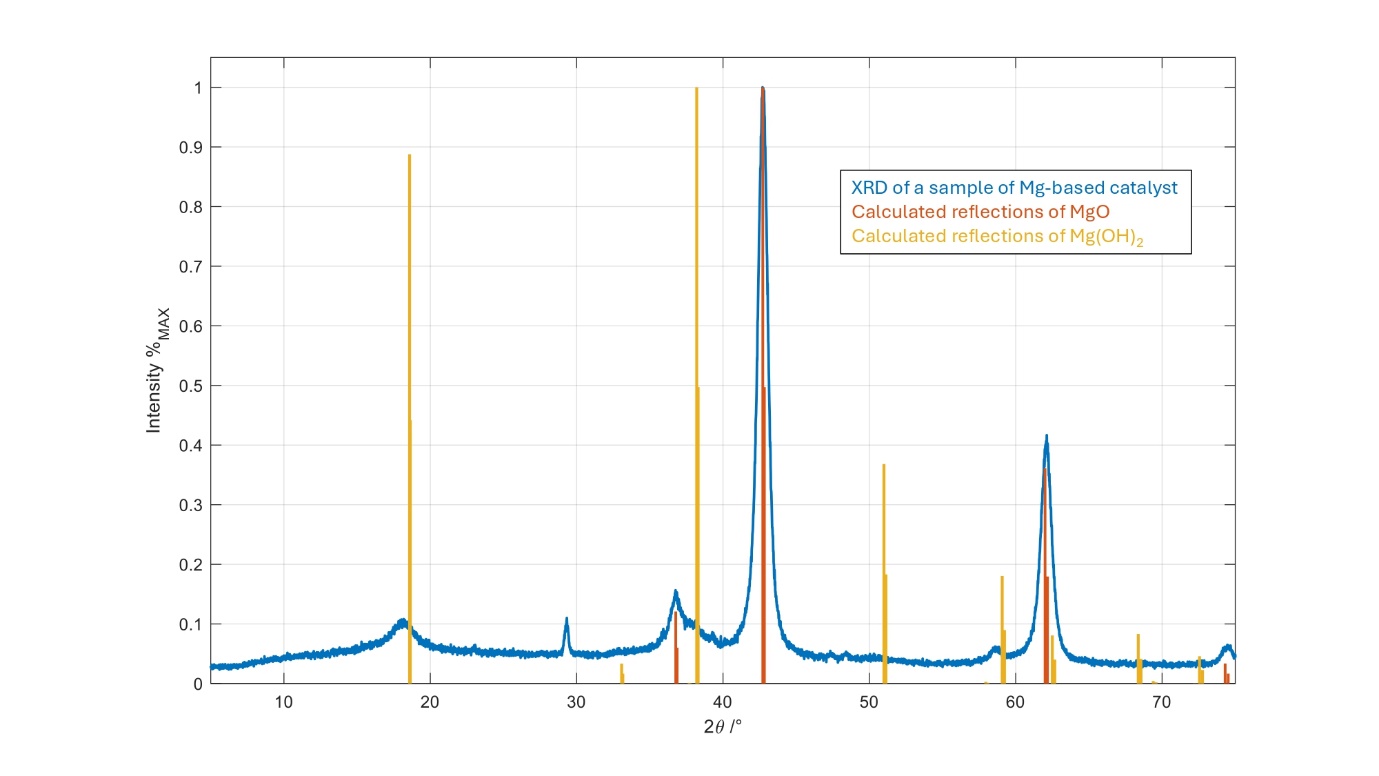


**Figure S5.** XRD analysis of the used MgO (blue line) compared with the calculated reflections of Mg(OH)_2_ (yellow line) and MgO (red line).

Table S1. Catalyst impact on temperature data for the reaction of 2.5 mmol of LGO with 6 mmol of dimethyl malonate at 10 W fixed power.

| Catalyst | Reaction time (min) | Max temperature (°C) |
| --- | --- | --- |
| Ca(OH)_2_ | 2 | 102 |
| Ca(OH)_2_ | 5 | 122 |
| Ca(OH)_2_ | 10 | 165 |
| MgO | 2 | 84 |
| MgO | 5 | 96 |
| MgO | 10 | 109 |
| NaOH | 2 | 92 |
| NaOH | 5 | 130 |
| NaOH | 10 | 136 |
| K_2_CO_3_ | 2 | 99 |
| K_2_CO_3_ | 5 | 158 |
| K_2_CO_3_ | 10 | 168 |
| KF/alumina | 2 | 89 |
| KF/alumina | 5 | 137 |
| KF/alumina | 10 | 153 |
| AlCl_3_ | 2 | 110 |
| AlCl_3_ | 5 | 161 |
| AlCl_3_ | 10 | 171 |
| FeCl_3_ | 2 | 102 |
| FeCl_3_ | 5 | 168 |
| FeCl_3_ | 10 | 173 |
| HNO_3_ | 2 | 82 |
| HNO_3_ | 5 | 114 |
| HNO_3_ | 10 | 124 |
| HCl | 2 | 83 |
| HCl | 5 | 104 |
| HCl | 10 | 118 |
| H_2_SO_4_ | 2 | 84 |
| H_2_SO_4_ | 5 | 128 |
| H_2_SO_4_ | 10 | 133 |
| ZnCl_2_ | 2 | 94 |
| ZnCl_2_ | 5 | 134 |
| ZnCl_2_ | 10 | 163 |
| CuCl_2_ | 2 | 81 |
| CuCl_2_ | 5 | 109 |
| CuCl_2_ | 10 | 121 |
| MnCl_2_ | 2 | 84 |
| MnCl_2_ | 5 | 107 |
| MnCl_2_ | 10 | 118 |
| SnCl_4_ | 2 | 81 |
| SnCl_4_ | 5 | 118 |
| SnCl_4_ | 10 | 157 |
| InCl_3_ | 2 | 93 |
| InCl_3_ | 5 | 149 |
| InCl_3_ | 10 | 158 |
| YbCl_3_ | 2 | 82 |
| YbCl_3_ | 5 | 105 |
| YbCl_3_ | 10 | 123 |
| Mont | 2 | 69 |
| Mont | 5 | 94 |
| Mont | 10 | 105 |
| K10 | 2 | 68 |
| K10 | 5 | 97 |
| K10 | 10 | 106 |
| AlCl_3_/Mont | 10 | 102 |
| AlCl_3_/Mont | 5 | 97 |
| AlCl_3_/Mont | 2 | 65 |
| AlCl_3_/K10 | 10 | 108 |
| AlCl_3_/K10 | 5 | 98 |
| AlCl_3_/K10 | 2 | 71 |
| SBA-15 | 2 | 68 |
| SBA-15 | 5 | 96 |
| SBA-15 | 10 | 108 |
| Amino SBA | 2 | 70 |
| Amino SBA | 5 | 100 |
| Amino SBA | 10 | 107 |
